# Biobanks in the low- and middle-income countries of the Arab Middle East region: challenges, ethical issues, and governance arrangements—a qualitative study involving biobank managers

**DOI:** 10.1186/s12910-022-00822-8

**Published:** 2022-08-14

**Authors:** Ahmed Samir Abdelhafiz, Mamoun Ahram, Maha Emad Ibrahim, Alya Elgamri, Ehsan Gamel, Rania Labib, Henry Silverman

**Affiliations:** 1 Department of Clinical Pathology, National Cancer Institute, Cairo University, Cairo, Egypt; 2 Department of Physiology and Biochemistry, School of Medicine, The University of Jordan, Amman, Jordan; 3 Department of Physical Medicine, Rheumatology and Rehabilitation, Faculty of Medicine, Suez Canal University, Ismailia, Egypt; 4 Department of Orthodontics, Pediatric Dentistry and Preventive Dentistry, Faculty of Dentistry, University of Khartoum, Khartoum, Sudan; 5 Department of Oral Rehabilitation, Faculty of Dentistry, University of Khartoum, Khartoum, Sudan; 6 Research Department, Children’s Cancer Hospital Egypt, Cairo, 57357 Egypt; 7 Department of Medicine, University of Maryland School of Medicine, 110 S. Paca Street, Baltimore, MD 21201 USA

**Keywords:** Biobanks, Arab Middle East region, Ethics, Informed consent, Data-sharing, Community engagement, Governance, Transparency, Trust

## Abstract

**Background:**

Biobanks have recently been established in several low- and middle-income countries (LMICs) in the Arab region of the Middle East. We aimed to explore the views of biobank managers regarding the challenges, ethical issues, and governance arrangements of their biobanks.

**Methods:**

In-depth semi-structured qualitative interviews were conducted with a purposive sample of eight biobank managers from Egypt (6), Jordan (1), and Sudan (1). Interviews were performed either face-to-face, by phone, or via Zoom and lasted approximately 45–75 min. After verbal consent, interviews were recorded and then transcribed. The authors performed a thematic analysis of the transcripts independently and then integrated the themes via a consensus process.

**Results:**

Biobank managers discussed the main challenges in establishing their biobanks. These included the staff’s lack of experience and training, limited funds, deficit awareness of biobanks, obtaining funding from different sources. Only four reported they were active in distributing biospecimens and health data to researchers. Six biobanks used a broad consent model, one used tiered consent, and another allowed participants to opt-out of being recontacted. Five managers avoided partnerships with pharmaceutical companies due to concerns with unfavorable reactions from the community. Five managers did not have clear policies for returning research results to the donors. Five expressed challenges with sample and data sharing with international collaborators; all five used material transfer agreements. The biobank managers revealed variable governance arrangements and activities with community involving awareness and educational efforts rather than active engagement. Several expressed the importance of transparency with the operations of their biobanks and gaining the trust of their stakeholders.

**Conclusion:**

Managers of biobanks in LMICs in the Arab Middle East encounter financial, operational, and social challenges toward their sustainability efforts. Discussions with key stakeholders are warranted to manage ethical issues involving informed consent, privacy, data sharing, and the return of results. We recommend that biobank managers in the Arab Middle East form collaborative networks within the region and internationally, develop trusting governance relationships with their stakeholders, and pursue engagement activities with their communities to enhance trust.

**Supplementary Information:**

The online version contains supplementary material available at 10.1186/s12910-022-00822-8.

## Introduction

The recent establishment of biobanks in many parts of the world has been instrumental in advancing genomic research and personalized medicine [1]. Biobank research has been prompted by several developments that include mapping the human genome and the ability to maintain large electronic databases that store vast amounts of information (i.e., big data). Types of biobanks include population-based (e.g., identification of environmental and genetic markers that contribute to disease susceptibility in populations), disease-centric (e.g., cancer and AIDS), and project-based (e.g., small-scale investigator-based research).

Biospecimens and health data collected and stored in biobanks for unspecified future research present challenges to the traditional practice of informed consent. Before the era of biobanks, investigators were obligated to give potential participants study-specific informed consent that included the purpose, risks, and benefits of the research, confidentiality methods for data security, and the nature of participants’ rights for every new study [2]. However, this consent model requiring research participants’ study-specific consent for each future study using their samples and data makes genomic studies involving secondary research more challenging to perform due to difficulties with recontacting participants. New consent models (e.g., broad consent, tiered consent, dynamic consent) provide more practical alternatives that would preclude the need to recontact and obtain consent from participants for each future but remain ethically problematic [3]. Other ethical challenges associated with biobank research involve methods to protect information privacy, data sharing, and the return of results [2].

Biobanks also face many financial, operational, and social challenges with their efforts to establish sustainability [3]. These challenges include developing a business model that rely on dependable funding sources, enhancing operational efficiency, and building trusting governance arrangements with researchers and potential donors [4, 5]. The establishment of trust requires methods to ensure transparency, accountability, and the active engagement of their communities [6, 7].

Several guidelines and best practices have been advanced to manage these challenges [8, 9]. However, variability in the political, institutional, and cultural conditions limits the seamless application of these guidelines in different settings [10], especially in low and middle-income countries (LMICs) [11]. As biobanking facilities are either underdeveloped or non-existent in many LMICs, the Biobank and Cohort Building Network (BCNet[12]) was established to allow LMICs an opportunity to work together in a coordinated and effective manner and jointly address the many challenges in developing sustainable biobanking research infrastructures [13].

Biobanks are emerging throughout the Arab Middle East. Biobanks have been established in several LMICs and high-income countries (HICs) in the Arab Middle East region. These include biobanks in Egypt, Jordan, and Sudan (LMICs) as well as those in Qatar and Saudi Arabia (HICs). There are, however, limited data regarding the specific nature of the challenges and ethical issues that these biobanks encounter. We aimed to explore the views of managers of several biobanks from LMICs in the Arab Middle East region regarding their challenges and ethical issues with the establishment and management of their biobanks. Our focus on biobanks from LMICs was predominantly due to the expectation that their challenges would differ from those established in HICs in the region.

## Methods

### Study design

We used an exploratory qualitative approach consisting of semi-structured interviews.

### Recruitment of participants

We recruited biobank managers between January 2020 and December 2020. These managers represented three LMICs in the Arab Middle East region. Managers who agreed to participate were invited to join a face-to-face, phone, or online interview.

### Sampling technique

We used a purposeful sampling technique based on our knowledge of biobank managers in the Arab Middle East. Such a sampling technique enabled us to identify individuals we knew would be knowledgeable and experienced in the field and would most likely be available and willing to participate in a study that involved discussion of sensitive issues. This sampling technique ensured the obtainment of information-rich cases [14]. Essentially, we were interested in achieving depth of understanding of the issues rather than generalizability of results, which is accomplished using quantitative methods.

### Procedures

We developed a semi-structured interview guide consisting of open-ended questions regarding several issues in biobanking. See Additional file 1 for the interview guide. We continually revised the interview guide to explore different emerging concepts raised by the discussion of prior participants.

Participants gave verbal consent prior to the interview. A repeat interview was conducted with the managers when necessary to clarify any section of the interview. The number of in-depth interviews was determined when we reached data “saturation” (i.e., redundancy of data collected) for the interviews.

We recorded the interviews conducted in Arabic and English, followed by a translation into English for the interviews conducted in Arabic and subsequent transcription. Each interviewer reviewed the transcript and the recording for accuracy.

### Analysis

We used the method of thematic analysis to identify themes [15]. This method consists of several steps. First: each interviewer read the transcripts line-by-line to familiarize themselves with the text and to develop codes. Themes were then generated both deductively, based on our prior analytic framework, as represented in the interview guide; and inductively, by allowing new themes to be considered from the text. The co-authors examined the themes for patterns until consensus was achieved on the final themes after several iterative discussions. The emergent themes were further compared with the available literature to ensure extrapolation of the results to similar research. One co-author performed a final verification step to ensure that the final themes represented a true reflection of the participants' statements on their biobanking practices. Codes and themes were organized using MAXQDA software [16].

## Results

Eight out of eleven managers we contacted agreed to participate in our research. Six were from Egypt and one each from Jordan and Sudan. All biobanks were either affiliated with universities or with non-profit organizations. All biobanks were disease-based biobanks, and the managers indicated that they collected samples from cancer or non-cancer patients to support genomic research and advance personalized medicine. The biobanks recruited donors from their associated clinics. Two of the biobanks were established more than 10 years ago, three had been in existence for 5–10 years, and three were established less than 5 years prior to this study. All biobanks except for one were active with collection activities from donors (biospecimens and associated health data). The relative immaturity of the biobanks in our sample is reflected by only four having policies to provide their biospecimens and health data to researchers either internal or external to their institutes.

Table 1 presents the themes and subthemes we identified from the interviews, several of which we discuss below.

**Table 1:** Identified themes and subthemes from the interviews

Themes	Subthemes
Goals of biobanks	Personalized medicine
	Knowledge of susceptibility to diseases
	Investigation of genetic diseases
Challenges to the establishment of biobanks	Lack of experience and training
	Limited start-up funding
	Lack of awareness and the novelty of the concept of biobanking
	Resistance from stakeholders
	Professional colleagues
	Patients and public
Sustainability	Definition of sustainability
	Funding from different sources
	Well thought-out business plans
	Sample utilization and collection of fees
	Collaborations with researchers
	Partnerships with the private sector
	International networking
Ethical issues	Types of informed consent
	Privacy and confidentiality
	Data sharing—Material Transfer Agreements
	Return of Results
Governance	Definition of Governance
	Types of Committees
	Sample Access Policies
	National Regulations
Community engagement trust	Activities to involve the community
	Concerns with public lack of trust
	Methods to enhance trust
	Transparency

### Challenges to establishment of biobanks

Biobank managers mentioned several challenges with the initial establishment of their biobanks. These challenges included the lack of experience and the need for training, limited start-up funds, lack of awareness and the novelty of the concept of biobanking, and resistance from stakeholders (professional colleagues and patients, and the public).

#### Need for training

All managers highlighted the importance of training. One manager reflected on this issue as follows:

The biobanking concept was not common in Egypt. So, the experience about how to establish the biobank, its equipment, ethics, and data protection were limited. The administrative procedures and financial issues were also challenging.

Six biobank managers reported that their staff received training from Europe or the USA institutions. The topics of the training programs included administrative aspects, sustainability, and ethical issues. One manager said:

The training I received in Luxembourg lasted for one month. part of it was theoretical about how to establish, how to manage, and how to sustain the biobank. Another part was about how to choose the software, and how to develop or collect a consent form. A third part was more practical about standard operating procedures (SOPs) and how to develop them for sample collection, processing, storage as well as retrieval.

#### Limited initial start-up funding

All biobank managers commented on the scarcity of initial start-up funding and capital resources; one manager said:

The main two issues are establishing the biobanking database and lack of resources. We are a charity hospital with limited resources, and it is costly to get the biobanking facilities and lab devices for research. All of these were challenges. We needed a competent person to allocate the money.

#### Lack of awareness and the novelty of the concept

Five managers reflected on the lack of awareness and the novelty of the concept of biobanks demonstrated by their colleagues. One manager said:

There is limited education about the importance of biobanking. Although we did have full support from the administration in our university, especially with our dean, we did not have full support at the university level. We had members from other faculties who did not think that establishing a biobank is important and that it is not a top priority for funding.

Another manager said:

The other challenge we faced was the physicians and how they were not very aware of the concept of biobanks.

#### Challenges from stakeholders

Four managers mentioned opposition to the establishment of biobanks from stakeholders representing their professional colleagues. One participant said:

We have communicated with different administrations at the institute; Some of them were supportive while others were not.

Another manager said:

We had an initial resistance from pathologists who always say that the tissue is their issue. They considered it as their baby. So, we have faced some difficulties with them.

Finally, a different manager said:

But there is somewhat a resistance from the part of the surgeons. They didn’t care. The medical oncologists are cooperative, but the surgeons’ position is not the same.

Three biobank managers mentioned that reluctance of patients to donate biospecimens and data represented another challenge to the establishment of biobanks. One participant said:

The main challenges we had were how to convince patients to donate their samples for research and how to explain their rights regarding the use of these samples.

Another manager mentioned:

Some patients think that donating for genetic research will help their physicians directly in managing their specific illnesses. When we explained that this is not available, some of them became very frustrated and reluctant to donate.

Managers reflected on patients not appreciating the importance of research or even being suspicious of the research activity. One manager said:

The biobank is still a new concept in scientific research and is not appreciated enough. The public can’t assess its importance…What we need from the community is the awareness of what’s going on instead of resisting or holding suspicious concepts regarding research.

When asked by the interviewer whether it was expected that there would be community resistance, the biobank manager said:

Yes, of course [the public] doesn’t feel easy with research. So, we need to raise their awareness.

Another manager said:

As I told you, they don’t understand ‘scientific research’. This is also a challenge we face that people do not know what research is. Some people think you will take the sample and they don’t trust us. We try to explain but sometimes they still refuse. We have also noticed that sometimes the consent and the signature scare them.

A manager expressed similar concerns:

Upper Egypt it is different from in Cairo or other cities. Culture-wise, they were not exposed before to the concepts of research and biobanks. They have no background on that. For example, a patient today refused to donate a sample for no reason. He read and understood the consent but decided not to donate. There are fears and concerns.

#### Sustainability

All managers discussed mechanisms to ensure the sustainability of biobanks. These included procurement of funding from different sources, development of a well-thought-out business plan, mechanisms for sample utilization and collection of fees, collaborations with researchers, partnerships with the private sector, and networking with international colleagues.

#### Funding sources

One manager’s general thoughts about sustainability were as follows:

Financial and social aspects are important. For financial sustainability, I should have quality samples to get the trust of the researchers, so I can get funding from funders. For social sustainability, there should be transparency to gain the people’s trust in dealing with the biobank.

Managers of biobanks mentioned procurement of funding from different sources. These included their hospitals, governmental bodies, local organizations, and collaboration with foreign partners. Two managers mentioned the receipt of funding from their hospital center. One said:

everything in the research department is funded by the hospital since they know the importance of biobanking.

One manager said regarding governmental sources and local and foreign organizations:

I can expect funding from government establishments, but I cannot imagine it from the NGOs except that they fund the researching projects.” Another said: “It was a donation given by the National Bank of Egypt.

One participant said:

Our fund came from the European Union to be used by our European partners and us.

Several managers mentioned applying for grants. One said:

We submit for grants, ‘calls’ for supporting the biobank. All monies sustained in the biobank are collected from the grants.

Another said:

But we have applied to get grants. We got grants for the equipment and supplies, liquid nitrogen.

One said:

we apply for a grant for the STDF (Science and technology Development fund). But we did not get accepted in this grant.

#### Business plans

None of the biobank managers had a current business plan regarding financial sustainability. One manager said,

No, we do not have a clear business plan. It is impossible to cover the entire cost. The goal is to cover our cost as much as we can.

#### Sample utilization and collection of fees

All managers mentioned that charging fees for the biospecimens and data would help support the operations of the biobanks. However, only four managers said they currently release samples to researchers for their projects. One said:

We have been releasing samples for research for 2 or 3 years now.

Another manager said:

A good solution is to provide specimens to funded researchers. A researcher with a grant has a priority because [at the end] I need to recover my cost. It will be difficult if the researcher does not have a grant.

Two managers, however, gave reasons why they do not charge fees for the samples. One said: "We feel that the concept is still relatively new for people. So, we do not want to be opposed to the idea that we sell samples. We may be misunderstood. So, we provide them for free. Another said: "We are a charitable institution. The hospital policy does not allow us to receive any money for the biobanking activities.”

#### Collaboration with researchers

Five managers discussed the importance of establishing collaboration with researchers who represent their customers. One manager said:

We want to collaborate with researchers from other well-known centers to have more well- designed data collections according to the needs of the research community.

Another manager highlighted the importance of providing high-quality samples to researchers, which enhances trust with researchers. One manager said:

Samples and data should be of high quality to get the trust of the researchers. So, we can receive more funding through participation in scientific projects to achieve financial sustainability.

However, one manager commented on a barrier to distributing samples to researchers external to the institution:

We need to respect the people’s doubts about giving the samples to researchers outside of the institute, so it is not that easy. So, we mainly work within the institute. We rarely collaborate with other research centers. Our priority is for the researchers inside the institute.

#### Partnership with the private sector

Relationships with the private sector represented a sensitive issue for five biobank managers, as many refused collaborations with pharmaceutical companies. One participant said:

Many pharmaceutical companies approached us to buy tissue samples, but we refused because we do not sell them. We think that this would be an ethical problem and the patients could refuse it. In addition, this would create a reputation about our center that it sells their tissues. We prefer that the tissue goes to research that is conducted by researchers from academia rather than industry.

Another manager echoed this viewpoint:

We have also mentioned that we won't release them to the pharma companies, because that would raise some issues regarding selling the samples. [We want] to stay away from troubles and just focus on research in pediatric cancer research and not on the global scope research.

Another manager said:

A partnership with a pharmaceutical company, at this stage- No, because it would raise a conflict of interest. Maybe in the future when looking for a treatment or management of certain conditions. That will be a very long way; that will be like the stage three or even four for our project and then, definitely without any hesitation.

Finally, another participant made a distinction between international and local pharmaceutical companies and said:

We hope that we will have collaborations with the local pharmaceutical factories rather than international ones in order to enhance also the local businesses, but not at this stage.

#### International networking

Four managers mentioned the importance of networking toward their efforts with sustainability. One manager said:

We need our personnel to attend the international conference to know what is going on. We are in a closed area; there are no biobanking conferences in our area. So, we need to attend events to improve our performance.

Another said:

First, more collaborations with other well-known centers, attending conferences, meeting with other centers.

When asked: “Have you ever thought of being a member of any regional or international biobank network organization as these networks might be beneficial for the development of the biobank?” the manager responded:

Absolutely, actually, this kind of networking is indispensable for me. And this is why I took it upon myself to reach out to University of Malaya and the University of Bergen. It is very important.

Another said:

It cost us a lot, but we need to be updated with all new techniques in improving the quality, so we can collaborate with higher centers in developed countries.

### Ethical issues

#### Types of informed consent

Six biobanks used broad consent when procuring samples and health data, one used a tiered consent for their ongoing research project at the time of the interview, and another allowed the participants to opt-out of being recontacted. One manager said:

We use broad consent, which gives us more flexibility and preserves the rights of the patient, that his/her data would remain confidential, and he/she has the right to withdraw at any time. All these details are written, and the patient becomes aware of these terms.

Biobank managers preferred broad consent rather than tiered consent since it obviates the participants' need to recontact. One manager commented on the difficulty of recontacting:

There is a problem in sustaining any research projects in our country, because it is extremely difficult to recontact the patients and I'm telling that from my experience on this issue.

#### Practices maintaining the privacy and ensuring confidentiality

Seven managers mentioned that privacy protection is maintained through reversible coding and limited access to samples and data from other individuals. One manager said:

The IRB makes sure that we have a policy of privacy and confidentiality and that we have tissues and samples that are coded and are linked to the medical record number of the patient in a secure place.

Another said:

Yes, we generate such codes using the biobanking software that links the biobank identity number to the donor’s medical number at the biobank. I think that we provide a high level of data security.

#### Challenges with sample and data sharing

Managers provided their viewpoints regarding sample and data sharing with investigators outside their institutions.

Several managers had an optimistic viewpoint regarding international collaboration. For example, one manager said:

We have been collaborating with researchers in the USA, as well as some Arab countries such as KSA [Kingdom of Saudi Arabia]. This has been controlled through MTAs with no restrictions. We participate as partners, and get academic recognition, since we participate in writing papers and in the analysis and are included in the publications as co-authors.

However, four managers from Egypt expressed concerns regarding international collaboration and benefit-sharing. One said:

There are concerns in the scientific community about the collaboration with the international organizations concerning the loss of sample control or benefit-sharing. Also, our law of scientific research restricts transfer of samples across borders except under certain conditions.

Another stated:

The most common comment we hear about this is that they will take our samples and conduct research on them. They may also make biological weapons.

Five managers mentioned using Material Transfer Agreements (MTA) when distributing samples to researchers. One said:

Anyone can request the samples. Although we rarely cooperate with another research center, we have a collaboration with New Giza Centers if they send a well-known protocols and policies. I never release samples without sending them to the committee that approves them. They then sign the material transfer agreement. Then they come to take the samples.

When asked about losing control over the samples, one manager said:

Yes, I know this incident with the Ebola research. Such concerns are possible if there is no material transfer agreement. But we are stringent about using one. All parties have to sign including the head of the institution that will receive the samples.

One manager said regarding the importance of an MTA:

There should be, of course a document that guides and controls the agreement between collaborators, so that if any violation happens, the document can be used as a legal proof of this violation. Collaborator usually sign a material transfer agreement in such collaborations, whether nationally or internationally.

Biobank managers also discussed the proper recognition of the primary investigators who produced the data when participating in international collaboration projects with high-income countries. One manager said:

If we will make an MOU, it should clearly provide that the biobank will be nominated in the acknowledgment. If a researcher is actively participating in the data collection, he will be part of the authorship. This should be clear.

#### Return of results

There were various opinions regarding the return of results to participants.

Five biobank managers said they do not have a clear policy regarding returning research results to participants. Also, one mentioned the lack of a robust Information Technology (IT) system:

Currently without an information system, it is not easy to handle the results using papers.

Biobank managers detailed slightly different approaches regarding the "return of results.” One manager said:

We mentioned in the consent that we won't recontact the participant and that the samples are specifically for the cancer research type.

In contrast, another manager said:

So, for the general biological data since we don't have results that will be meaningful for our participants; we don't tend to tell them, and we will state that clearly in our consent that there is actually nothing no information that will be beneficial for you. But when we further extend the research for specific diseases, we give patients the option, so we ask them part of the consent that we are providing ‘Do you want to know about any about the results of any possible test for the samples that we collected from you?’

Five managers mentioned they would communicate results that have a clinical value to the participants. One said:

Basically, I think that mostly we do not return data unless the IRB indicates that the data might be important to return. We have studied BRCA 1 and 2 in some breast cancer patients and we informed the patients of the results. So, they can tell their relatives whether they would like to be checked or not, and some of them have come to be checked.

Another said:

I must then explain that the research made on your samples is not necessarily related to your disease and if it is related to your case, we will send to you with the results.

Another manager mentioned that they allow participants an opportunity to opt-in:

We give the patient an option to get research results as part of the consent form. Their responses will determine whether we will provide them with the results.

### Governance

#### Definitions

Biobank managers held slightly different concepts of governance. For example, several equated governance with,

Management that encompasses processes, structures and arrangements upon which individuals or groups are given the authority to achieve the desired results.

Another manager said:

Governance is about how to make decisions, who solves the problems, and who follows up the biobank progress.

Similarly, another said:

The governance is the responsible persons or the individuals that manage the biobank. For example, in the institute, there is ethical governance, scientific governance, and administrative governance. In the institute, we have an IRB committee that we take its approval and the scientific committee to determine the scientific rationale of any research proposal. We also have an administrative committee. Besides, we have a director that puts day-to-day goals and objectives. He then suggests them to the scientific committee. We have finally a release committee that decides to give the samples or not.

Two managers defined governance in terms of the structural hierarchy of the biobank. For example, one manager said:

The biobank governance is managed by a hierarchical structure that is overseen by the Scientific Medical Advisory committee, IRB, the hospital administrators, and a biobank committee that consists of 8 to 9 members. They are the heads of the key stakeholders’ departments like pathology, surgery, researchers.

#### Types of committees

All managers mentioned the role of committees in their governance structure. However, the types and number of committees were variable between the biobanks. For example, institutional review boards (IRBs) were present in all biobanks, and it was the only committee that provided oversight to the various functions of three biobanks. One manager said:

No, it is just the IRB. It also serves as a scientific committee. They review projects so that the samples are only released for important projects, they ask PIs for modifications until they approve it.

Several managers mentioned the presence of other committees, such as a scientific committee and a biobank committee, to oversee the decisions of the biobank. One manager said:

We have an IRB committee and a scientific committee that determines the scientific rationale of any research proposal. We also have an administrative committee, and a release committee that decides to give access to the samples or not.

Four managers mentioned sample access committees that controlled researchers' access to biospecimens and data. The managers described the process of sample access. For example, one manager said:

To get access to samples, the proposal must be scientifically sound and approved by the scientific committee. However, before that, the researcher must check for the availability of samples. There could also be competing proposals. Therefore, each proposal is given a score, and then it is decided which one gets a priority. This is followed by IRB approval.

Another manager said:

Actually, there is a release policy, or a workflow of samples release in the cancer hospital. So, if a researcher wants samples from our biobank, he should study the diseases that he wants to get tissue samples for. He must then go to the Medical Advisory Committees (MAC) since they have a short application form. It includes questions about the sample size and type as well as his study aims. If they approve his application, they will release samples to him. So, the matter is based on the study aims.

#### National regulatory structure

In response to being asked whether having committees are enough, one manager said:

If there is a clear law, it would be easier because all these are individual efforts. We have put our SOPs that are approved by the Institution. But there is nothing that provides that the SOPs fulfill the local policies and laws. Actually, there are almost no laws.

### Community engagement

Biobank managers held different concepts of community engagement. Three biobank managers mentioned activities related mainly to community interaction rather than actual engagement. For example, one said:

These activities are limited; For example, our biobank staff made some initiatives to communicate with the community. The cancer hospital tries to promote scientific research and how it directly affects the treatment results. Other than this, there are no other efforts.

Other managers mentioned that their efforts were mainly focused on enhancing awareness and education of the community in biobank activities. For example, one participant said:

At our institute, we have explained the concepts to people working in it. We have assessed the ‘Knowledge, Attitude, and Practice’ regarding the biobank before and after training. We have uploaded some presentations about biobanking on the institute website and to the Facebook page of our department. We need to communicate more with the community members, and to reach them using social media.

When asked whether the community should be involved with the policies regarding consent, one manager said:

I think that the consent form and policies should be approved by the ethical committee and the Faculty of Medicine. There should be community members in the ethics committee, and in other committees as well.

However, another manager responded:

In the work of the biobank? No, the community has no role. Like what? What could be the role of the community? What type of support do we need from the community? And what will you present back to the community?

### Trust

#### Trust concerns

Managers expressed concerns with potential donors lacking trust in the biobanking activities. One manager said:

Sometimes patients are unsure where these samples would go. Although the consent explains everything, they still have no trust.

Another manager mentioned the need to demonstrate that the donors’ samples were put to good use:

I think the key to trust is based on the achievement, not just collecting samples even if they do not understand the impact of the research, they need to see a successful example.

#### Methods to enhance trust

Six biobank managers mentioned the importance of establishing and maintaining trust with their stakeholders, especially with the public/patients. Methods included promoting awareness, and communication, avoiding relationships with pharmaceutical companies, and avoiding charging fees for samples.

One manager mentioned the value of awareness in gaining trust. He said:

Awareness, a lot of awareness, about research in general and biobanking in particular. I do not think people in all of Egypt have heard about biobanks.

Another manager stressed the communication of results:

As I told you, many engagements in seminars will raise their awareness and make them feel its benefit for the patients. So, if we have good findings, we should announce them.

Biobank managers mentioned other mechanisms to establish trust with the community. For example, one manager said:

It requires continuous communication with the stakeholders in the community. Reach out to different stakeholders and try to conduct seminars to those stakeholders using very simple language, telling them what the biobank actually does and why we need them. Stakeholders are anyone who has a voice in the community, it could be the Imam in mosques, or the priest in churches, so they need to be aware, so that when someone asks for their religious opinions about donating, they know how to guide people to do. All of these stakeholders need to be informed of the activities of the biobank.

Regarding relationships with pharmaceutical companies:

There are no plans for it. They contact me about this. But this is not the right time to collaborate with pharma companies since the public could misunderstand the biobank concept. I think it should be postponed until we gain public trust.

#### Transparency

Biobank managers also mentioned the importance of transparency in establishing trust. One manager said:

We must be transparent with donors. In general, for example on our website all details about what we work on and progress of operations.

Another manager said:

Because we have transparency from the first step from the handling of data and storage of data and all other procedures and follow all standards in the industry and this will build trust between us.

Four biobank managers mentioned efforts to establish transparency with the funders, donors, and the community. For example, one manager said:

All of our guiding principles, SOPs, from where we receive the samples and to whom we share them. All of these documents must be clear and available for anyone to see, whether they be for the donors or researchers or the community in general. In addition, announcing the results of the research studies done on the samples is another way of ensuring transparency, that the samples are put for good use.

## Discussion

Our study revealed significant insights regarding challenges, ethical issues, and governance mechanisms with biobanks in several LMICs in the Arab Middle East region. We discuss several of these as follows.

Biobank managers mentioned fundamental challenges to the process of establishing their biobanks. These included lack of training, limited start-up funding, lack of awareness and the novelty of the concept of biobanking, and resistance from stakeholders.

The lack of experience and training represented a challenge with the initial start-up efforts of biobanks. The operations of biobanks require the presence of highly specialized and qualified personnel to manage the whole life cycle of samples beginning with the collection, processing, storage, and ending with their release to researchers [17]. Although the past 20 years have witnessed the development of many biobanking educational programs, most of these programs reside in Western countries. Local, regional, and national networks can facilitate and coordinate several educational activities to help and support new biobanks in the region. One example of these networks is the Egyptian Biobank Alliance, which was created to coordinate and support biobanking activities in Egypt [18].

Limited knowledge of stakeholders about biobanks in the Arab Middle East region has been reported in the literature [10, 19–21]. Educational activities aimed at communicating the concept of biobanks have been limited and often fail to reach many stakeholders [22]. Lack of knowledge about biobanks has been shown in other parts of the world. For example, Klinsgler and colleagues reported that a third of researchers in Germany reported inadequate knowledge about the existence of biobanks at their institutions and limited knowledge about the types of samples provided by these biobanks [23].

The biobank managers in our study mentioned resistance from their peers to support the biobanking activities and the unwillingness of potential donors to donate to the biobank. A recent survey involving the public in several Arab countries in the Middle East showed that more than 80% had misperceptions regarding biobanks associated with an unwillingness to donate biospecimens to a biobank [24]. This reluctance to participate in biobank research compares with previous surveys conducted in the Arab Middle East demonstrating a low percentage (< 30%) of the public who would agree to participate in clinical trials [25–27]. A recent study regarding the public's attitudes in Egypt, Jordan, and Saudi Arabia toward participating in Covid-19 clinical trials showed that only 15–17.5% of the surveyed people were willing to participate in such research [28].

Achieving sustainability represented another challenge for biobanks. The basic principle of sustainability includes attention to the three-pillar concept of sustainability development. These dimensions include financial, operational, and social issues [3]. Financial aspects include being knowledgeable of local, regional, and international funding sources. Operational sustainably relates to the efficiency of biobank activities, including professional management and proper use of samples. Social sustainability relates to the trustworthiness and hence, the acceptability of biobanks by different stakeholders [29]. All of these dimensions need to be pursued with equal priority. An example of such an approach includes the application of sustainability at Biobank Graz, where its sustainability plan included project management and business planning and involved governance and ethical, legal, and social issues [28].

Financial aspects represent a demanding issue to achieving sustainability, as biobanks strive to maintain their activities against continual and increasing costs. Accordingly, biobanks should have a business plan to guide their future activities. However, all of our biobank managers lacked a business plan, which might signify the field's immaturity in the region.

Despite the absence of a business plan, financial support can be achieved through institutional assistance, external grants, user fees, and providing different services to their clients [30].

Several of our managers indicated the receipt of funding from their institutions as well as from external sources. Many indicted having plans for cost recovery through providing services and user fees. Although user fees are generally accepted worldwide [31], they might face resistance in the Arab region, where researchers expect to obtain samples at no cost. Not surprisingly, one of the participants reported that researchers who obtained access to their samples negotiated to pay less for the samples they received. A user fee calculator developed by biobanks in Canada can provide solutions to these problems by developing fair and realistic user fees for biobanks in the region [20].

Although user fees help biobanks achieve financial stability, the extent to which researchers' utilization of samples and services could represent another challenge [19]. A survey of 276 biobanks from different parts of the world showed that the utilization rate of samples was 10% or lower in more than half of the participating biobanks [21]. Biobanks should have effective marketing and communication strategies with researchers to understand their needs, promote the services of the biobank, and review the policies for samples and data access on a regular basis [17, 19]. A more specific method to attain higher sample utilization rates involves understanding the perspectives of potential users of biobanks— i.e., researchers who use biospecimens in their research projects. For example, a recent survey involving potential users in Germany showed that approximately half of the respondents were not aware of the services of the respective local biobank [23]. Other respondents stated that “the samples required were not available, the costs were too high and information about the available biospecimens was not readily accessible.” Other efforts to elicit the attitudes of such stakeholders toward biobanks have been performed in the UK [32] and the Netherlands [33]. However, long-term financial sustainability cannot be achieved by applying user fees alone. Biobanks should apply a holistic approach by relying on mixed funding streams including governmental funding, research institutions' funding, and funding by research grants [34].

Partnerships with private companies, e.g., pharmaceutical companies, represent a potential source of income for biobanks. However, such partnerships have engendered much debate. Commercialization raises issues about fairness and benefit-sharing [35], and most of our participants did not support such partnerships as it might engender mistrust among their stakeholders, particularly the public. The issue involves a public concern with selling biospecimens, which represents commodification. These concerns are reasonable for several reasons. First, the public might see partnerships with industry as signifying biobanks are in the business of selling biospecimens. The commodification of body samples might prove to many as lacking moral value. Knowledge of such partnerships among the public might compromise the reputation of the biobanks, which might outweigh the potential financial benefits from it.

Partnering with pharmaceutical companies might also affect trust with stakeholders [19, 36, 37], leading to public reluctance to donate their samples and health data to biobanks. Trust in the community can easily be lost if biobanking activities involve partnering with industry [38]. Commercialization should be an issue for a broad community discussion with relevant stakeholders, including the public, researchers, institutional administrators, and policymakers, to reach a consensus on guidelines about this sensitive issue. An ethical framework has been recommended to guide sharing biospecimens and health data with industry that emphasizes transparency and autonomy [39].

Establishing collaborative ties with local and international researchers represents another important step to achieving sustainability. However, prior research indicates that underutilization of biobank resources is a common problem for biobanks and a source of ethical concern [40, 41]. For example, clinical research laws in some Arab countries, such as Egypt, prevent any form of trading in samples [18]. Furthermore, several managers conveyed their concerns regarding the transport of samples to international researchers. For example, Egyptian biobank managers expressed apprehensions about international collaboration, including fears of the samples being used to develop biological weapons. Although such fears have not been reported among physicians in Egypt [10], policymakers harbor concerns related to national security considerations. As required by the recent Egyptian clinical research law, the entry or exit of human samples for medical research is allowed only after obtaining the approval of the Supreme Council for ethical review of clinical medical research [42].

Biobank managers from the other countries in our study did not harbor similar concerns with international data sharing. Commentators have stressed that global sharing of samples and data should be a priority [12, 43]. Also, underutilization of biospecimens with potential collaborators can inevitably impact the sustainability of biobanks. Challenges with international sample and data sharing can be rectified through coordinated and planned efforts [44].

Mechanisms should also be established to prevent the biopiracy of samples and the exploitation of participants. Material Transfer Agreements (MTAs) and Data Transfer Agreements (DTAs) represent a framework whenever samples are exported outside of the country to ensure fair benefit sharing, prevent repurposing of samples [43], and appropriate recognition of host researchers. However, only four biobank managers in our study mentioned the use of such agreements when samples are exported internationally. Essential components to be included in MTAs have been identified [45], although some recommended items have proved to be problematic [46]. When sharing samples, unambiguous MTAs with precise specifications on how individuals' and communities' rights and interests can be protected are imperative. When sharing specimens and data from individuals in LMICs, MTAs should also outline how benefits from any therapeutics or vaccines produced by the research will be shared with the LMICs.

International biobanking networks have evolved and can be helpful with fostering sustainability [3, 47]. The African Union established a sustainable model that builds on existing structures to establish a network of biobanks that accelerate the development, evaluation, and research on the diagnostics required for disease control and prevention programs [48]. In 2013, the WHO established the formation of the Low- and Middle-Income Countries (LMIC) Biobank and Cohort Network (BCNet). BCNet aims to provide a platform for collaboration between the international community to support biobanking and cohort-building activities and develop sustainable biobanking research infrastructures to facilitate the collection of high-quality biological samples for research, using best practice principles and guidelines [13].

Biobank managers discussed several types of ethical issues related to biobanks. One involved the type of informed consent. Broad consent was the preferred model for Arab biobanks. Studies suggest that participants may be supportive of broad consent if done respectfully, e.g., community engagement is considered a pre-requisite for the use of broad consent [49]. However, another option includes tiered consent that provides more autonomy, as participants indicate their specific preferences regarding the types of future research where their samples will be used and how data will be shared with other parties [50]. A previous study showed that many Egyptians favored a consent model that restricts future research conducted on their samples to their illness only, which resembles the tiered consent approach [37]. A recent study involving several Arab Middle East countries demonstrated that the public equally favored broad consent and tiered consent models [24]. Tiered consent might engender more trust with biobanks as public members might prefer to have more control over the types of research that will use their specimens and data. Tiered consent has also been preferred in other African countries [51]. Dynamic consent represents another model in which donors can choose their preferences about sample use through web-based interfaces [52]. This type of consent is controversial, primarily due to costs that might make it prohibitive in LMICs.

Return of results represents another ethical concern. Many of the biobanks in our study did not release samples to researchers and as such, these biobanks managers in our study reported that they currently do not have a clear policy to return results. Among the biobanks that used biospecimens in research projects, some indicated that results would not be returned while others would return results if they would have a clinical effect. Return of results is considered a right for sample donors by stakeholders in the different Arab countries [53]. However, several concerns are associated with the return of results. One involves what should be returned and the methods of returning results. These issues are significant in the genomic era, where many results may be complex and difficult to interpret [56]. A survey of Dutch investigators revealed that many believed that individual genetic results should be returned only if these results have a clinical effect, such as the possibility of prevention of a disease or access to treatment according to the available clinical standards of care on prevention or treatment [57].

Stakeholders in the Arab Middle East countries should deliberate and develop clear policies regarding managing the return of genetic results. A helpful resource includes the H3Africa Consortium, which recently released guidelines on this issue [54]. Their recommendations include that "feedback of results" should include those findings that are 1) "robustly associated with disease causation," i.e., high clinical validity, and 2) "should be able to guide therapy or prevent disease," i.e., clinical utility. The Consortium also recommends that feedback on individual genetic research findings be accomplished by medical genetic health professionals. As individuals with such expertise might be lacking in many LMICs, biobanks should develop the means to train other healthcare professionals with the capabilities to communicate with participants about individual genetic research results. Finally, the Global Alliance for Genomics and Health has developed a "2021 Policy on Clinically Actionable Genomic Research Results" that should also serve as a reference for policy development [55].

Participants in our study established variable governance structures, similar to the variability of governance of biobanks reported in South Africa [56]. Establishing an efficient governance framework for biobanking research was among the significant challenges raised by research ethics committee members from 18 African countries [2]. Governance mechanisms, broadly construed, consist of all formal and informal policies, processes, and structures that guide the activity of a biobank. However, governance goes beyond the management of workflow processes that lead to decisions. Good governance also includes transparency, accountability, broad-based participation, empowerment of stakeholders, and regulatory oversight [5]. All these components lead to the development of trust [2, 5]. .Establishing trust is an essential element of good governance and is associated with achieving the long-term goals of biobanks [6]. Transparency, defined as the "provision of relevant and useful information in a timely, engaging, and understandable fashion," contributes to accountability, which leads to increased public trust in biobanks [6]. If biobanks expect the public to donate their biospecimens and data, then there needs to be transparent knowledge regarding how data are governed, protected against privacy breaches, shared with third parties, linked with other data sets, and the kinds of research that uses the data sets, and the results of the research studies are communicated to the stakeholders. Transparency of these components leads to trust that encourages the public to participate in biobanking activities. Absent such trust relationships, biobanks will find it difficult to recruit donors and collect samples. Indeed, trust is vital for the effective functioning of biobanking and hence, represents an essential component for effective governance and sustainability.

The active engagement of the community in the operations of biobanks is essential for establishing trust [44]. Obtaining public trust encourages a donation to the biobank and public funding [45]. If the community becomes suspicious of the governance structures of biobanks, or if there is a general perception that biobanks do not support public needs and interests, biobank activities may be significantly hindered [46].

Our study showed that biobanks adopted a limited “engagement” with the community that consisted only of enhancing awareness and education. Stakeholders in South Africa have reported a lack of engagement between biobank researchers and potential participants [56]. Active community engagement should go beyond mere awareness campaigns and should involve hearing their voices about issues related to biobank governance [7]. Community engagement is a continuous process and is not limited to a one-time meeting with community members [49]. Biobanks should try to engage the communities targeted by the research with all activities of the research process, from the initial phases of planning and data collection until the project leads to results or changes in policies or recommendations [49]. Communities should be involved in the governance structures of biobanks. Such partnership will support research activities and provide transparent oversight for ethical governance of the biobank to ensure proper use of samples and data for the best of the community [7]. Such engagement allows both parties to express their needs and concerns and will promote trust in research and biobanking activities. Through this type of engagement, the interests of current and future participants are well recognized and handled [57].

This engagement can be done in different ways, including community advocates and forums and community advisory boards [52, 53]. An interesting example of community engagement is the one that involved the United States biobanks in the eMERGE Network. The biobanks in this network used different methods to engage the community, including focus groups, educational presentations, mail, surveys, and website information. This engagement allowed biobanks to modify their educational strategies, processes, and protocols [44]. Biobanks in the region could learn from these examples to develop better engagement programs that best fit the local culture and knowledge of the local community. Proper governance of biobanks must involve the community in developing clear protocols regarding these issues, including detailing the process of deidentification or anonymization of the samples and data prior to sharing [43].

## Limitations

We recognize several limitations in our study. First, most participants were from Egypt, and only a few were from other countries. This sampling, however, reflects the distribution of biobanks in the region and provides a wide-ranging account of the challenges of biobanks in this region. Based on our exploratory analysis, we recommend a follow-up quantitative study to accurately obtain the extent of the practices occurring throughout the LMICs in the Arab Middle East. Second, biobanks in this study were in different stages of development, which affected participants' responses to some questions (e.g., sample access policies and international collaborations issues) and accounted for the demonstrated variable practices. Finally, this study did not fully explore the concept of benefit sharing with international collaborations, probably due to the early stages of most of the biobanks.

## Conclusions and recommendations

Biobanking activities are growing in the Arab Middle East region. The field is still in the maturation phase, and many face challenges common to other LMICs. While financial and operational issues are essential in the success and sustainability of biobanks, the social aspects involved with establishing trust represent a key component. Trust requires transparency and accountability mechanisms and active engagement with the communities. All of these factors are needed for good governance arrangements. We recommend that biobank managers in the Arab Middle East form collaborative networks within the region and internationally, develop trusting governance relationships with their stakeholders and donors, and pursue active engagement with their communities. Consensus over debatable ethical issues is also needed to build frameworks and guidelines that can support biobanking activities in the region. This strategy will improve the willingness of potential donors to participate, encourage the public to be involved in different issues related to biobanking, and promote the future sustainability of biobanks.

## Supplementary Information

**Additional file 1.** Interview guide for policy makers of biobank centers.

## Data Availability

The datasets used and/or analyzed during the current study are available from the corresponding author on reasonable request. Due to the small sample size and the concern with identification of the participants in this study, we do not provide a link to the data set so that privacy can be maintained.
