# Supplementary material for: Biobanks in the low- and middle-income countries of the Arab Middle East region: challenges, ethical issues, and governance arrangements—a qualitative study involving biobank managers
Source: BMC Med Ethics. 2022 Aug 14;23:83. doi: 10.1186/s12910-022-00822-8 (PMC9375918; doi:10.1186/s12910-022-00822-8)
Supplement: Supplementary file 1 — Additional file 1. Interview guide for policy makers of biobank centers. [file 12910_2022_822_MOESM1_ESM.pdf]

Interview guide for policy makers of biobank centers

Informed consent: explain the purpose of the interview/research, discuss confidentiality issues, discuss that there might be societal benefits but no direct individual benefits. Ask for their verbal consent (if you have asked your REC that verbal consent is acceptable), if not have them send you an email that they give consent.

Purpose: Explain that you will ask specific questions about their biobank and their thoughts regarding biobanking issues. Interview will last approximately 1 hour and there are no right or wrong answers.

1. Tell me about your biobank.
   LOOK FOR:
   a. Length of time in existence
   b. Type of biobank. For example, academic, private, non-profit, etc.
   c. Type of biobank: hospital-integrated/disease based/population based
   d. What are the goals of your center's leaders when they established the biobank?

2. What have been the challenges with establishing and maintaining your biobanks

3. Who do you consider to be your stakeholders? Look for: funders, public, and researchers

4. How is It funded? **GRANT/PRIVATE/GOVERNMENTAL/PUBLIC/COMMERCIAL**
   a. What are the funding issues with establishing of your biobanks?
   b. Is there private funding?
   c. Have you obtained grants to help fund the biobanks?

SUSTAINABILITY

5. Tell me about plans for sustainability in the future?
   Financial
   a. Will sustainability depend on continued grant funding 5 years from now?
   b. cost recovery of its running costs?
   c. Cost recovery and commercialization models are being considered, which may create barriers for researchers in LMICs.
   d. ASK IF THEY HAVE A BUSINESS PLAN

   Operational: quality of specimens and datasets

   Social: public acceptance

6. What are your plans for utilization of the specimens? Is there a concern with decreased utilization?
   a. Look for: formal relationships with other universities
   b. Fees?

7. Tell me about research at your center.
   a. Types of research is conducted.
   b. Does it include genetic research on biological samples?

c. Kind of patient population

8. What are the expectations regarding doing research that involves biospecimens?

9. What type of training have you received? The staff?

10. Tell me about the REC
    a. What challenges do they face?
    b. HOW DID THE REC FEEL ABOUT PATIENTS BEING ASKED FOR CONSENT FOR FUTURE UNSPECIFIED RESEARCH?
    c. Community members on the REC
    d. Do you think the REC members are up to date with biobank concepts?

11. Why do patients donate? What are their expectations?

12. Have you encountered any issues with patients refusing to donate? Why do they refuse?

    Are there fears that research may test for stigmatizing illnesses?
    Are there fears that samples may exported abroad?
    Are there fears due to lack of data security/re-identification?

COMMUNITY ENGAGEMENT

13. Are there particular community concerns about the use of blood samples?
14. How do you establish trust with the community?

    a. Do they think that engagement with the community can enhance trust? Such engagement can give the community greater decision making over the use of their samples, implement fair benefit sharing and enhance governance.

    b. Lack of community engagement has been criticized as presenting the public with as passive recipients of knowledge while implying that science has the "right" answers.

15. Is the community involved in the management of the biobank? For example, advisory board, developing processes for recruitment, informed consent and policies of the biobank?

16. Do you inform the community of the results and if so, how?

Informed Consent

17. What type of informed consent do patients give when they are asked to donate samples?
    a. Inquire whether they use a broad consent model (patients give consent to any type of unspecified future research). Are their concerns as to whether broad consent represents a valid consent, because one does not know what research will be conducted.
    b. Instead, does the biobank use a tiered consent model (in which patients are given a range of options to include broad consent model, re-contact for consent for any future research, or given a choice as to whether they give consent for studies that

involve only their disease category, or consnet for studies that involve unrelated health conditions.

GOVERNANCE

18. Tell me about the governance of your biobank.

Governance: The framework of rules and practices by which the biobank ensures accountability, fairness, and transparency to patients, researchers, and society.

Robust governance processes are needed to ensure that research is carried out efficiently, effectively and ethically.

Governance frameworks typically include

- Data Access Committees (DAC),
- Ethics Review Boards,
- scientific committee,
- data (informatics) committee
- specimen quality committee

- Inquire about model of biobank: ownership or custodian (have they thought about this concept even been discussed?)

- Inquire about the committees that ensure accountability to their policies.

- How does the biobank ensure transparency and accountability to those who donate samples?

- What performance metrics do they intend to provide to their funders?

TYPES OF POLICIES
INVOLVNG DONORS

a. Informed consent process
b. Privacy and confidentiality of the specimens and data?
c. Ensuring data security?
d. policies regarding returning results to donors
e. Policies regarding commercial aspects/profits?

Data Access Committee? How is access regulated? Have they shared samples yet?
What are the challenges with sample/data access?

Oversight boards?

Challenges with exporting samples/data to institutions.
Ask about MTA

Have other investigators from outside the center requested specimens?
Are there concerns with utilization?

INTERNATIONAL – DATA SHARING

Challenges with international collaboration with exporting samples/data to other countries?

Samples? Data?

If collecting samples from patients with Covid, are there intentions to share with others?

Concerns can include fears of losing control of the samples after export, issues regarding ownership of the samples, fairness with sharing of benefits that results from the research, appropriate academic recognition of researchers in publications. In essence, is there mistrust of international collaborators?

Need to collaborate internationally

- there needs to be a recognition that the richness of biobank use over time will depend on linkages with health data, genomic data and other multi-omics data integration and analyses.
- Data sharing and biobanking have the potential to improve the quality and value of research.

A good governance regarding sharing samples and data internationally include:

- Collaboration/engagement
- Fairness in benefit sharing/credit
- Capacity building

Fairness: Does the biobank desire capacity building efforts from international collaborators when they access the samples/data?

a. Examples include technology and infrastructure needed to do sample analysis and training of local personnel with specialized skills to conduct, analyze and publish studies.

Are there national regulations?

Are you a member of a local/regional/international biobanks network/organizations? If the answer is yes, how do you benefit from this?

Do you have any relationships/partnerships with pharmaceutical companies?

a. If not, do you plan to have such relationships in the future?
b. Do you think such relationships can contribute to sustainability?
   THIS IS AN IMPORTANT QUESTION TO ASK BECAUSE ONE OF THE GOALS OF GENETIC RESEARCH IS TO ENHANCE PERSONALIZED MEDICINE, I.E. TAILOR DRUGS TO THE GENETIC PROFILE OF THE PATIENTS.

2. Close by asking whether he/she has any other issues that needs to be explored.
